# Power Amplification Increases With Contraction Velocity During Stretch-Shortening Cycles of Skinned Muscle Fibers

**DOI:** 10.3389/fphys.2021.644981

**Published:** 2021-03-31

**Authors:** André Tomalka, Sven Weidner, Daniel Hahn, Wolfgang Seiberl, Tobias Siebert

**Affiliations:** ^1^Department of Motion and Exercise Science, University of Stuttgart, Stuttgart, Germany; ^2^Human Movement Science, Faculty of Sports Science, Ruhr University Bochum, Bochum, Germany; ^3^School of Human Movement and Nutrition Sciences, University of Queensland, Brisbane, QLD, Australia; ^4^Human Movement Science, Bundeswehr University Munich, Neubiberg, Germany; ^5^Stuttgart Center for Simulation Science, University of Stuttgart, Stuttgart, Germany

**Keywords:** contractile behavior, muscle stretch, muscle shortening, muscle damping, mechanical power, performance enhancement, eccentric contractions

## Abstract

Muscle force, work, and power output during concentric contractions (active muscle shortening) are increased immediately following an eccentric contraction (active muscle lengthening). This increase in performance is known as the stretch-shortening cycle (SSC)-effect. Recent findings demonstrate that the SSC-effect is present in the sarcomere itself. More recently, it has been suggested that cross-bridge (XB) kinetics and non-cross-bridge (non-XB) structures (e.g., titin and nebulin) contribute to the SSC-effect. As XBs and non-XB structures are characterized by a velocity dependence, we investigated the impact of stretch-shortening velocity on the SSC-effect. Accordingly, we performed *in vitro* isovelocity ramp experiments with varying ramp velocities (30, 60, and 85% of maximum contraction velocity for both stretch and shortening) and constant stretch-shortening magnitudes (17% of the optimum sarcomere length) using single skinned fibers of rat soleus muscles. The different contributions of XB and non-XB structures to force production were identified using the XB-inhibitor Blebbistatin. We show that (i) the SSC-effect is velocity-dependent—since the power output increases with increasing SSC-velocity. (ii) The energy recovery (ratio of elastic energy storage and release in the SSC) is higher in the Blebbistatin condition compared with the control condition. The stored and released energy in the Blebbistatin condition can be explained by the viscoelastic properties of the non-XB structure titin. Consequently, our experimental findings suggest that the energy stored in titin during the eccentric phase contributes to the SSC-effect in a velocity-dependent manner.

## Introduction

The most common form of muscle action during terrestrial locomotion is characterized by eccentric muscle action (active lengthening) immediately followed by concentric muscle action (active shortening). Such stretch-shortening cycles (SSCs) occur in both cyclical and non-cyclical locomotion at the level of the muscle-tendon unit (Komi, 2000; Ishikawa et al., 2005; Aeles and Vanwanseele, 2019; Navarro-Cruz et al., 2019) and the muscle fiber (Gillis and Biewener, 2001; Nikolaidou et al., 2017). So far, a large number of experiments at the level of the muscle-tendon unit have shown an increase in muscle force, work, and power output during the shortening phase of SSCs compared with pure shortening contractions (Cavagna et al., 1968; Bosco et al., 1981; Gregor et al., 1988; Seiberl et al., 2015). This SSC-effect (i.e., increased muscular performance) is further associated with amplified muscular efficiency accompanied by reduced metabolic energy consumption (Cavagna et al., 1968; Holt et al., 2014). The underlying mechanisms of the SSC-effect that have been discussed in the literature include activation dynamics, contributions of stretch reflexes, storage and release of elastic energy, and history-dependent effects of muscle action associated with residual force enhancement (rFE) (Van Ingen Schenau et al., 1997; Cormie et al., 2011; Seiberl et al., 2015).

In contrast to a large number of SCC studies at the level of the muscle-tendon unit, there are only a few studies at the level of the skinned muscle fibers that examined the SSC-effect in the sarcomere itself (Fukutani et al., 2017a; Fukutani and Herzog, 2019, 2020a; Tomalka et al., 2020). An SSC-effect of about 30% of work enhancement relative to pure shortening was reported for slow stretch-shortening velocities [about 10% maximum shortening velocity (*v*_0_)] and long muscle fiber lengths [2.4–3.3 μm, descending limb of the force-length relation (*F*-*l*-r)] of skinned rabbit soleus muscle fibers (Fukutani et al., 2017a; Fukutani and Herzog, 2019). Tomalka et al. (2020) demonstrated that the SSC-effect in skinned rat soleus muscle fibers also occurs in the more physiological working range (plateau region and ascending limb of the *F*-*l*-r) at fast contraction velocities (∼85% *v*_0_). Mechanisms related to activation dynamics, reflex activity, and elastic recoil from tendons—as discussed for *in vivo* muscle action—cannot be responsible for the SSC-effect in skinned muscle fiber experiments. Thus, an additional mechanism needs to be found within the sarcomere itself. Using Blebbistatin for XB-inhibition, Tomalka et al. (2020) further showed that both XBs- and non-XB structures contribute to the SSC-effect on the skinned muscle fiber level, which is supported by other studies (Fukutani et al., 2017a; Fukutani and Herzog, 2020a).

These findings nicely fit into our current understanding of eccentric muscle action, as there is experimental evidence that non-XB structures like titin also account for increased forces during the stretch (Leonard and Herzog, 2010; Tomalka et al., 2017) and after the stretch (rFE) (Herzog, 2018; Freundt and Linke, 2019; Tahir et al., 2020). By definition, rFE describes the phenomenon of increased steady-state isometric forces after active muscle lengthening compared with the corresponding force during a purely isometric contraction (Abbott and Aubert, 1952; Edman et al., 1982). Several model approaches (Rode et al., 2009; Nishikawa et al., 2012; Schappacher-Tilp et al., 2015; Heidlauf et al., 2017) and experimental evidence of physiological mechanisms (Herzog et al., 2014; Mártonfalvi et al., 2014; Rivas-Pardo et al., 2016; Dutta et al., 2018) support the idea that titin plays an essential role in active muscle force generation (for recent reviews, see Linke, 2017; Nishikawa, 2020; Fukutani et al., 2021). Moreover, it is known that titin is a viscoelastic protein that interacts with the XBs, stores energy, and preserves force upon muscle stretch (Bianco et al., 2007; Chung et al., 2011; Herzog et al., 2014). Based on recent findings, it is assumed that the mechanisms of enhanced force generation triggered by muscle stretch are the same as triggered during the stretch-phase of SSCs and thus contribute to the SSC-effect on the level of the muscle fiber (Fukutani and Herzog, 2019; Tomalka et al., 2020).

Further, Tomalka et al. (2020) suggested that XB-kinetics are related to the SSC-effect at the single skinned muscle fiber level by XB-cycling that allows titin-actin interactions (Astier et al., 1998; Nagy, 2004; Bianco et al., 2007; Dutta et al., 2018; Li et al., 2018; Powers et al., 2020). Additionally, the elastic energy stored in elongated XBs during active muscle stretching (Huxley et al., 1994; Wakabayashi et al., 1994) might contribute to the SSC-effect during shortening (Cavagna et al., 1981). However, while only the XBs can produce active force, titin’s viscoelastic behavior is likely to contribute to the SSC-effect (by storage and release of elastic energy) in a velocity-dependent manner (Freundt and Linke, 2019).

Therefore, variations in XB and non-XB kinetics with increasing stretch velocity associated with a decrease in XB-binding and detachment of bound XBs (muscle “give”) (Katz, 1939; Flitney and Hirst, 1978; Choi and Widrick, 2010) might have an impact on the SSC-effect. Consequently, the influence of different stretch-shortening velocities on mechanical work and power output in the SSC should be examined. Thus, experiments in the physiological range (along the ascending limb to the plateau region) of the *F*-*l*-r with different ramp velocities (30, 60, and 85% *v*_0_) and the same ramp lengths (lengthening, shortening) were performed in this study to characterize the velocity dependence of the SSC-effect. The different contributions of XB- and non-XB structures to single skinned muscle fiber force and performance amplification were identified using the XB-inhibitor Blebbistatin. We hypothesize that the SSC-effect decreases with increasing SSC-velocity due to the force-velocity-relationship (*F-v-*r) on single muscle fiber force. Thus, the muscle forces, mechanical work, and power output should decrease in a non-linear manner as a function of increasing SSC-velocity.

## Materials and Methods

### Preparation, Handling, and Experimental Setup

**FIGURE 1:**
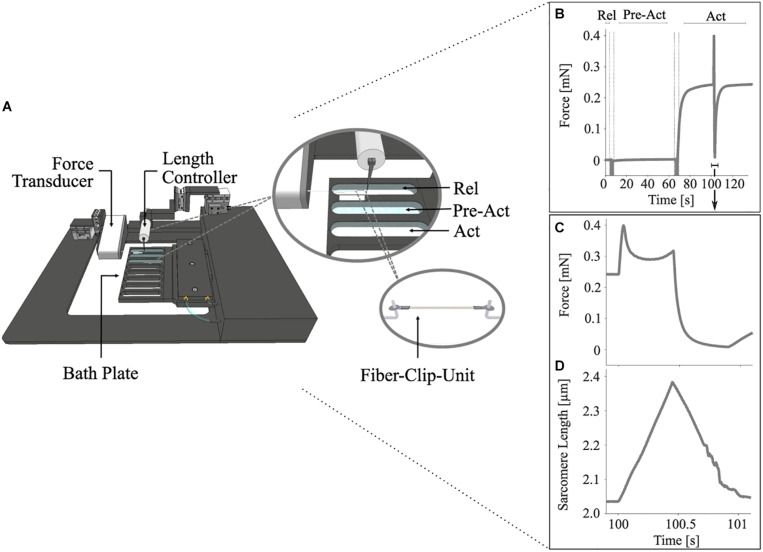
Schematic diagram of the permeabilized fiber apparatus **(A)** and a diagram showing the control experiments’ protocol **(B–D)**. **(A)** Close-up cut-away view of the eight-well bath plate (top right) showing three chambers filled with solutions and a single fiber mounted onto the hooks with T-clips (bottom right). **(B)** Representative force-time trace showing the changes in muscle force during an entire stretch-shortening cycle (SSC) with previous pre-activation (Pre-Act) and relaxation period (Rel) (*n* = 1, raw, unfiltered data). Enlarged views of the force response **(C)** and the change in sarcomere length **(D)** for an SSC with 85% maximum contraction velocity at supramaximal activation level (Act; *p*Ca = 4.5). Each of these experiments consists of an isometric phase, then a ramp transient, and then an isometric phase. The force is shown in mN, and the sarcomere length is recorded at maximal activation and is shown in μm. All ramp experiments have been conducted at a constant stretch/shortening magnitude of 0.17 *L_*S*_/L_*S0*_* on permeabilized single fiber segments from rat soleus muscles at 12°C experimental temperature. The vertical lines indicate the time for solution change. The arrow indicates the onset of activation.

Muscle preparation, storage, and activation techniques for permeabilized single muscle fibers were in line with Tomalka et al. (2017, 2020). Briefly, experiments were performed on glycerinated skinned single fiber segments from soleus muscles of six freshly killed female Wistar rats (age: 3 months, weight: 428–520 g, cage-sedentary, 12–12 h light: dark cycle, housing-temperature: 22°C). The skeletal muscle fibers from rats used for this study have been provided by another animal study approved according to the German animal protection law [Tierschutzgesetz, §4 (3); Permit Number: 35-9185.81/0491]. The small bundles (50–100 fibers) were stored in a storage solution (see section “Solutions”) containing 50% glycerol at –20°C for 4–6 weeks. As previously described, single fibers were prepared before the experiment (Tomalka et al., 2020). The fibers were treated with a relaxing solution (see section “Solutions”) containing Triton X-100 (1% v/v) for 1–2 min at 4°C to chemically disrupt the muscle membranes without affecting the contractile apparatus (Fryer et al., 1995). Afterward, a fiber segment 0.8–1.2 mm long was cut from the fiber, and T-shaped aluminum clips were mounted at its extremities for attachment between the lever arms of a high-speed length controller (322 C-I, Aurora Scientific, Canada) and a force transducer (403a, Aurora Scientific, Canada) (Figure 1A). The two ends of the fiber were fixed with glutaraldehyde in rigor solution and glued to the clips with fingernail polish diluted with acetone (Burmeister Getz et al., 1998). The length (*L*), width (*w*), and height (*h*) of the fiber were measured at 0.1 mm intervals in the central segment of the relaxed fiber with a 10 × dry-objective (NA 0.30, Nikon) and a 10× eyepiece. The individual sarcomere length (*L*_*S*_) was set to 2.4 ± 0.05 μm, which is within the optimal sarcomere length (*L*_*S0*_) range for maximal isometric force (*F*_0_) development in the activated state (*p*Ca 4.5) (Stephenson and Williams, 1982). The fiber cross-sectional area was determined assuming an elliptical cross-section of single muscle fibers (π*h*w/4) and was 4,844 ± 1,246 μm^2^ (mean ± standard deviation). The *L*_*S*_ was measured using a high-speed camera system (901B, Aurora Scientific, Canada) in combination with a 20 × ELWD dry-objective (NA 0.40, Nikon) and an accessory lens (2.5×, Nikon).

### Experimental Protocol

Stretch-shortening cycle experiments comprised two conditions of repeated measurements. The control condition (Figures 1B–D) was designed to investigate the dynamic total force response during varying isovelocity SSCs. The experiments with the XB-inhibitor (Blebbistatin condition) repeated the control condition and suggested the contribution of non-XB elements to force production during isovelocity SSCs. Each SSC experiment consisted of an isometric phase, followed by a ramp phase (lengthening and shortening) and a final isometric phase (Figure 1B).

For the control condition, single skinned fibers (six rats, 14 fibers) were activated at ∼2.0 μm *L*_*S*_, stretched to *L*_*S0*_ of ∼2.4 μm [in the activated state (*p*Ca 4.5)], and then immediately shortened to ∼2.0 μm *L*_*S*_ with varying stretch-shortening velocities of 30, 60, and 85% *v*_0_ in a randomized order.

An identical protocol was repeated for the same skinned muscle fibers (five rats, 13 fibers), in the presence of 20 μmol l^–1^ Blebbistatin in all solutions (see section “Solutions”) to identify non-XB contributions to muscle force (Cornachione and Rassier, 2012; Shalabi et al., 2017; Tomalka et al., 2020). Blebbistatin is a photosensitive chemical that blocks the force-generating transition of the bound actomyosin complex from a weakly to a strongly bound state and causes myosin heads to bind to actin without exerting any isometric force (Iwamoto, 2018; Tomalka et al., 2020). Thus (a major population of) XBs remain in the pre-power-stroke state weakly attached to actin (Minozzo and Rassier, 2010; Rahman et al., 2018). Blebbistatin does not affect titin mobility (Shalabi et al., 2017).

Only the data of one muscle fiber were discarded due to insufficient inhibition of force generation capability by Blebbistatin (remaining active isometric force at *L*_*S0*_ > 20% *F*_0_).

The average active isometric force at optimum sarcomere length *L*_*S0*_ was 0.29 ± 0.08 mN, while the mean optimum muscle fiber length was 0.77 ± 0.09 mm. The isometric force corresponds to relative average stress of 60.04 ± 9.49 kPa. The maximum shortening velocity of the skinned soleus muscle fibers from adult male Wistar rats was 0.46 ± 0.13 *L*_0_ s^–1^ (*n* = 6), and the curvature factor of the force-velocity-relation was *curv* = 0.07 ± 0.02. In separate experiments, the fiber specific *v*_0_ was calculated based on our experimental data from six to eight isotonic contractions against forces in the range of 0.1 *F*_0_ to 0.9 *F*_0_ (two fibers each from two rats and one fiber each from two other rats).

To preserve the structural and mechanical properties in maximally activated fibers over a longer period and to reduce sarcomere inhomogeneities, the “cycling protocol” by Brenner (1983) was used. To ensure the structural and mechanical integrity of fibers in the experiments, the following criteria were applied to discard fibers: (1) isometric force in reference contractions was decreased by more than 10%; (2) aberrant behavior of force-traces, evidenced by artifacts, oscillations, or abrupt flattening was noted; and (3) lesions, ruptures, or fiber contortion were identified visually. For the determination of force degradation, isometric reference contractions at *L*_*S0*_ were performed before and after each ramp contraction. In the ramp experiments (control condition), the isometric force in successive activations decreased at an average rate of approximately 3.2% per activation. All experiments were conducted at a constant temperature of 12 ± 0.1°C. At this temperature, the fibers proved very stable and were able to withstand rapid ramp perturbations over an extended period as well as prolonged activations (Ranatunga, 1982, 1984; Bottinelli et al., 1996; Tomalka et al., 2017).

### Calculations of XB- and Non-XB Forces

To separate XB and non-XB forces during SSCs, the forces obtained during the Blebbistatin condition were subtracted from the forces obtained during the control condition. This rather simple method was used previously (Tomalka et al., 2017) and is based on the following assumptions: First, XBs produce a constant average force during isokinetic stretch after an initial equilibrium of XB-distributions (Huxley, 1957; Huxley and Simmons, 1971). Second, Blebbistatin suppresses the active XB-based forces to a negligible level. This was assessed by comparing the initial isometric force at 2.0 μm *L*_*S*_ with and without XB-inhibitors. Administering Blebbistatin suppressed active XB forces by 98%, so it can be expected that Blebbistatin suppresses active forces during SSCs to a similar extent. Third, is assumed that Blebbistatin does only affect the active XB-based force production during SSCs (see section “‘Isolated XB’ Forces During the SSC”).

### Solutions

The relaxing solution contained (in mM) 100 TES, 7.7 MgCl_2_, 5.44 Na_2_ATP, 25 EGTA, 19.11 Na_2_CP, and 10 GLH (pCa 9.0). The preactivating solution contained (in mM) 100 TES, 6.93 MgCl_2_, 5.45 Na_2_ATP, 0.1 EGTA, 19.49 Na_2_CP, 10 GLH, and 24.9 HDTA. The activating solution contained (in mM) 100 TES, 6.76 MgCl_2_, 5.46 Na_2_ATP, 19.49 Na_2_CP, 10 GLH, and 25 CaEGTA (pCa 4.5). The skinning solution contained (in mM) 170 potassium propionate, 2.5 MgCl_2_, 2.5 Na_2_ATP, 5 EGTA, 10 IMID, and 0.2 PMSF. The storage solution is the same as the skinning solution, except for the presence of 10 mM GLH and 50% glycerol (v/v). Cysteine and cysteine/serine protease inhibitors [*trans*-epoxysuccinil-L-leucylamido-(4-guanidino) butane, E-64, 10 mM; leupeptin, 20 μg ml^–1^] were added to all solutions to preserve lattice proteins and thus sarcomere homogeneity (Linari et al., 2007; Tomalka et al., 2017). pH (adjusted with KOH) was 7.1 at 12°C. 450 U ml^–1^ of CK was added to all solutions, except for skinning and storage solutions. CK was obtained from Roche (Mannheim, Germany), and Blebbistatin was obtained from Enzo Life Sciences Inc. (NY, United States). All other chemicals were obtained from Sigma (St. Louis, MO, United States).

### Data Processing and Statistics

**FIGURE 2:**
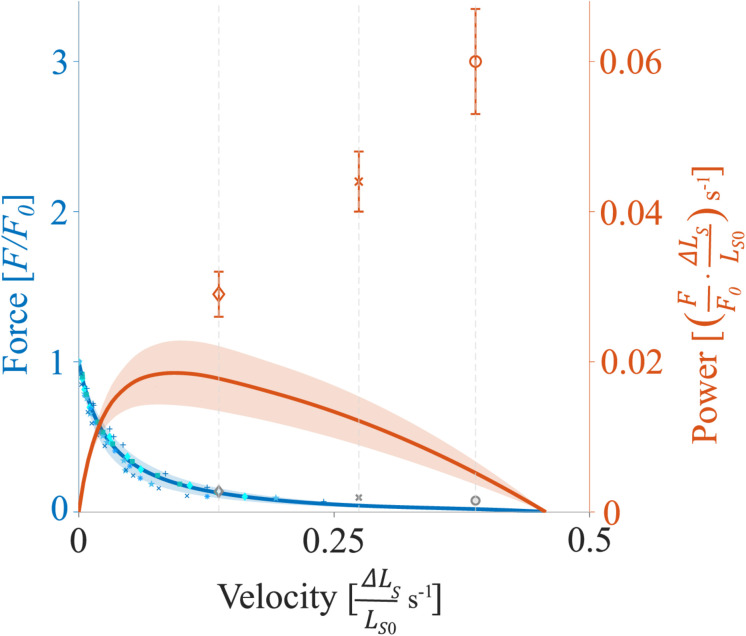
Representative force-velocity-relationship (*F-v*-r) and power-velocity-relationship (*P-v*-r) for soleus muscle. The blue solid line (and underlying individual data points) indicates the mean *F-v*-r for shortening contractions (positive velocities), while force declines as a function of shortening velocity (two fibers each from two rats and one fiber each from two other rats). The orange solid line indicates the mean *P-v*-r for shortening contractions. Power is maximum at intermediate shortening velocities at about 0.09 $\frac{\Delta \,L_{S}}{L_{S\,0}}$ s^− 1^ (∼20% *v*_0_). The shaded regions around the solid lines indicate the corresponding standard deviations (SD). Orange diamonds, crosses, and circles (mean ± SD) indicate maximum power output obtained during the shortening phase of SSCs with 30% *v*_0_, 60% *v*_0_, and 85% *v*_0_, respectively. Gray diamonds, crosses, and circles indicate the mean calculated XB-based forces (section “Contributions of ‘Isolated XB’ Force to Work and Power as a Function of Velocity”) obtained during the shortening phase of SSCs at respective velocities. *F*_0_ is the maximum isometric muscle force, velocity is given in relative units ($\frac{\Delta \,L_{S}}{L_{S\,0}}$ s^− 1^), and power is reported as relative values $(\frac{F}{F_{0}}\cdot \frac{\Delta \,L_{S}}{L_{S\,0}}$) s^− 1^.

Data were collected at 1 kHz with real-time software (600A, Aurora Scientific, Canada) and an A/D Interface (600A, Aurora Scientific, Canada). A custom-written MATLAB (MathWorks, Natick, MA, United States) script was utilized for data analysis. Unless stated otherwise, forces are expressed in absolute values (mN) or normalized to the individual maximum muscle force (*F/F_0_*). The shortening velocity is reported in relative units (Δ*L*_*S*_/*L*_*S0*_ s^–1^) or normalized to the fiber specific maximal shortening velocity (*v/v_0_*). Fiber lengths are expressed relative to the optimum fiber length (*L/L_0_*). Sarcomere lengths are expressed relative to the optimum sarcomere length (*L_*S*_/L_*S0*_*) or are reported in absolute values (μm). Mechanical work was calculated as the line integral of the changing force over the entire shortening distance during the SSCs and the pure shortening contractions and is expressed in relative values$(\frac{F}{F_{0}}\cdot \frac{\Delta \,L_{S}}{L_{S\,0}}$). The power output as a function of velocity (*P-v-*r, orange solid line of Figure 2) was calculated based on the force-velocity-relation for shortening contractions (*F*-*v*-r, blue solid line of Figure 2). Power is reported as relative values $(\frac{F}{F_{0}}\cdot \frac{\Delta \,L_{S}}{L_{S\,0}}$) s^–1^ or normalized to the maximal individual power (*P/P_0_*). The maximum mean power output *P*_0_ was 0.018 ± 0.005 $(\frac{F}{F_{0}}\cdot \frac{\Delta \,L_{S}}{L_{S\,0}}$) s^–1^ at 20% *v*_0_.

The power output of single skinned muscle fibers as a function of velocity calculated during isovelocity SSCs with varying velocities ranging from 30 to 85% *v*_0_ results from the work that was done within a particular time, delta [Δ] *t*:

$$\frac{F}{F_{0}}\cdot \frac{\Delta \,L_{S}}{L_{S\,0}}\cdot \frac{1}{\Delta \,t}$$

To avoid ambiguity and misinterpretations of the terminology of “positive work” and “negative work” in the following text, they are defined as follows: positive work is performed when an active muscle shortens by internal forces. Negative work is performed when an active muscle lengthens by external forces (Cavagna et al., 1965).

All data are presented as mean + standard deviation (SD) unless stated otherwise. To test whether the work (and power) significantly differs between the two conditions (control vs. Blebbistatin), a repeated-measures ANCOVA (factor 1 phase and covariate velocity) was used. Significant differences in the forces at the end of the stretch (control vs. Blebbistatin) were tested using a paired *t*-test. To test for differences in the energy recovery (defined as the ratio of elastic energy storage and release in the SSC; *W_*con*_/W_*ecc*_*) between both conditions, a paired *t*-test was used on data pooled across velocities in respective condition. To test whether the work and power output differ between the varying SSC velocities (30%, 60%, and 85% *v*_0_), a two-way repeated-measures ANOVA (factor 1 velocity and factor 2 phase) was calculated. In case that the ANOVA demonstrated significant main effects, *post hoc* analyses were performed using the student’s *t*-test with Bonferroni correction. The statistical tests were likewise performed for the control and the Blebbistatin experiments and a comparison of both conditions. The level of significance was set at *p* < 0.05. Statistical analyses were realized using SPSS 26 (IBM Corp., Armonk, NY, United States). The effect sizes of Cohen’s *d* were calculated as $d=\frac{M_{1}-M_{2}}{S_{\text{pooled}}}$, where *M* is the mean and *S*_*pooled*_ = S⁢D1 2+S⁢D2 22 (Cohen, 1988). The effect sizes were classified as small (*d* = 0.2), medium (*d* = 0.5), and large (*d* = 0.8) (Cohen, 1988).

## Results

Significant differences between the control and the Blebbistatin condition (*p* < 0.001) were observed for all parameters tested. Negative mechanical work during the stretch [control: −0.20 ± 0.01 vs. Blebbistatin: −0.04 ± 0.01 $\frac{F}{F_{0}}\cdot \frac{\Delta \,L_{S}}{L_{S\,0}}$], positive mechanical work during shortening [control: 0.03 ± 0.00 vs. Blebbistatin: 0.01 ± 0.00 $\frac{F}{F_{0}}\cdot \frac{\Delta \,L_{S}}{L_{S\,0}}$], negative power output during the stretch [control: −0.32 ± 0.13 vs. Blebbistatin: 0.07 ± 0.04 $(\frac{F}{F_{0}}\cdot \frac{\Delta \,L_{S}}{L_{S\,0}}$) s^–1^], positive power output during shortening [control: 0.04 ± 0.01 vs. Blebbistatin: 0.02 ± 0.01 $(\frac{F}{F_{0}}\cdot \frac{\Delta \,L_{S}}{L_{S\,0}}$) s^–1^], and force at the end of the stretch were larger in the control condition (1.16 ± 0.20 *F*_0_) compared with the Blebbistatin condition (0.42 ± 0.14 *F*_0_). In contrast, energy recovery (*W_*con*_/W_*ecc*_*) was significantly lower in the control condition (−0.15 ± 0.02) compared with the Blebbistatin condition (−0.25 ± 0.06).

### Control Condition: Effects of Velocity on Mechanical Work and Power Output

**FIGURE 3:**
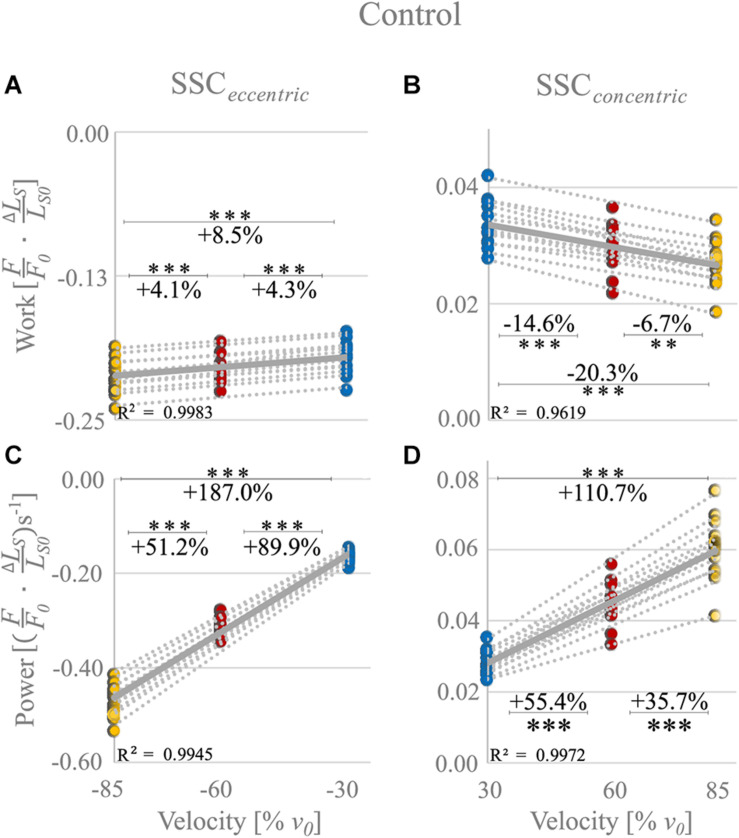
Control condition. Influence of varying stretch-shortening velocities on work and power during the lengthening phase (SSC_eccentric_; negative work/power; **A,C)** and the shortening phase (SSC_concentric_; positive work/power; **B,D)** of SSCs, respectively. The gray dotted lines of the scatterplots shown in **(A–D)** indicate the individual paired data values, and solid gray lines indicate the mean values (*n* = 14 fibers from six rats). The blue circles indicate the SSC at (±)30% *v*_0_, the red circles at (±)60% *v*_0_, and the yellow circle at (±)85% *v*_0_. Work/power values are expressed in relative values $(\frac{F}{F_{0}}\cdot \frac{\Delta \,L_{S}}{L_{S\,0}}$) and $\left(\frac{F}{F_{0}}\cdot \frac{\Delta \,L_{S}}{L_{S\,0}}\right)$s^− 1^, respectively. Asterisks mark differences in work/power with varying velocities in the intergroup comparison. Significance levels are marked as follows: ^∗∗^*p* < 0.01 and ^∗∗∗^*p* < 0.001. The control condition refers to the experiments without XB-inhibition.

The negative mechanical work during the stretching phase of the SSCs, when work is done on the muscle, increased significantly (+8.5 ± 2.5%, *p* < 0.001, *d* = 1.19, *R*^2^ = 0.99) with increasing stretch velocity (Figure 3A and Table 1).

**TABLE 1:** Descriptive statistics and pairwise comparisons of work and power values obtained during the control experiments.

Control								

				Pairwise comparisons work $[\frac{F}{F_{0}}\cdot$$\frac{\Delta \,L_{S}}{L_{\mathrm{S0}}}]$				*p*-values
	
Descriptive statistics				Mean differences	SD	95% Confidence interval of the difference		
		
Variable	Mean	SD				Lower	Upper	
W*_*ecc*_* 30% *v*_0_	−0.195	0.014	W*_*ecc*_* 30% *v_0_* −W*_*ecc*_* 60% *v*_0_	−0.008*	0.005	0.003	0.013	<0.001
W*_*ecc*_* 60% *v*_0_	−0.204	0.013	W*_*ecc*_* 60% *v_0_* −W*_*ecc*_* 85% *v*_0_	−0.008*	0.005	0.004	0.013	<0.001
W*_*ecc*_* 85% *v*_0_	−0.212	0.014	W*_*ecc*_* 30% *v_0_* −W*_*ecc*_* 85% *v*_0_	−0.017*	0.005	0.012	0.021	<0.001
W*_*con*_* 30% *v*_0_	0.034	0.004	W*_*con*_* 30% *v_0_* −W*_*con*_* 60% *v*_0_	−0.005*	0.001	0.004	0.006	<0.001
W*_*con*_* 60% *v*_0_	0.029	0.004	W*_*con*_* 60% *v_0_* −W*_*con*_* 85% *v*_0_	−0.002*	0.002	0.000	0.003	0.008
W*_*con*_* 85% *v*_0_	0.027	0.004	W*_*con*_* 30% *v_0_* −W*_*con*_* 85% *v*_0_	−0.007*	0.002	0.005	0.009	<0.001

				**$\text{Power}[(\frac{F}{F_{0}}\cdot$$\frac{\Delta \,L_{S}}{L_{S\,0}})s^{1}]$**				

*P*_*ecc*_ 30% *v*_0_	−0.164	0.012	*P*_*ecc*_ 30% *v_0_* −*P*_*ecc*_ 60% *v*_0_	−0.147*	0.010	0.138	0.157	<0.001
*P*_*ecc*_ 60% *v*_0_	−0.312	0.020	*P*_*ecc*_ 60% *v_0_* −*P*_*ecc*_ 85% *v*_0_	−0.160*	0.014	0.146	0.173	<0.001
*P*_*ecc*_ 85% *v*_0_	−0.471	0.032	*P*_*ecc*_ 30% *v_0_* −*P*_*ecc*_ 85% *v*_0_	−0.307*	0.021	0.287	0.327	<0.001
*P*_*con*_ 30% *v*_0_	0.029	0.003	*P*_*con*_ 30% *v_0_* −*P*_*con*_ 60% *v*_0_	0.016*	0.003	−0.019	−0.013	<0.001
*P*_*con*_ 60% *v*_0_	0.044	0.006	*P*_*con*_ 60% *v_0_* −*P*_*con*_ 85% *v*_0_	0.016*	0.004	−0.020	−0.012	<0.001
*P*_*con*_ 85% *v*_0_	0.060	0.009	*P*_*con*_ 60% *v_0_* −*P*_*con*_ 85% *v*_0_	0.032*	0.006	−0.037	−0.026	<0.001

Work/power values ± SD are expressed in relative values$(\frac{F}{F_{0}}\cdot \frac{\Delta \,L_{S}}{L_{S\,0}}$) and ($\frac{F}{F_{0}}\cdot \frac{\Delta \,L_{S}}{L_{S\,0}}$) s^–1^, respectively. *W*, mechanical work; *P*, power output; *ecc*, eccentric phase during active muscle stretch; *con*, concentric phase during active muscle shortening; %*v*_0_, percentage of maximum contraction velocity; *ns*, not significant. *The mean difference is significant at the 0.05 level.

Negative work was significantly larger for fast (−85% *v*_0_) compared with moderate (−60% *v*_0_) stretching velocities (+4.1% ± 2.2%, *p* < 0.001, *d* = 0.61; yellow vs. red circles of Figure 3A) and for moderate compared with slow (−30% *v*_0_) stretching velocities (+4.3% ± 2.7%, *p* < 0.001, *d* = 0.63; red vs. blue circles of Figure 3A).

For the shortening phase of the SSCs, the positive mechanical work, when work is done by the muscle, decreased significantly (−20.3% ± 5.9%, *p* < 0.001, *d* = 1.19, *R*^2^ = 0.96) with increasing shortening velocity. Positive work was significantly smaller for moderate compared with slow velocities (−14.6% ± 4.2%, *p* < 0.001, *d* = 1.28; Figure 3B) and for fast compared with moderate shortening velocities (−6.7% ± 5.6%, *p* < 0.01, *d* = 0.51; Figure 3B). The mean negative work output during muscle stretch was about seven times the amount of positive work during muscle shortening of SSCs (Table 1).

The negative power output increased successively with increasing stretch velocities (+187.0% ± 6.7%, *p* < 0.001, *d* = 12.88, *R*^2^ = 0.99) for the stretching phase of the SSC (Figure 3C). Negative power was significantly larger for fast compared with moderate stretching velocities (+51.2% ± 3.2%, *p* < 0.001, *d* = 6.05; Figure 3C) and for moderate compared with slow stretching velocities (+89.9% ± 4.9%, *p* < 0.001, *d* = 9.12; Figure 3C).

For the shortening phase of the SSCs, the positive power output increased significantly (110.7% ± 15.6%, *p* < 0.001, *d* = 4.89, *R*^2^ = 0.99) with increasing shortening velocity. Power was significantly larger for moderate compared with slow shortening velocities (+55.4% ± 7.6%, *p* < 0.001, *d* = 3.36; Figure 3D) and for fast compared with moderate shortening velocities (+35.7% ± 8.1%, *p* < 0.001, *d* = 2.18; Figure 3D).

**FIGURE 4:**
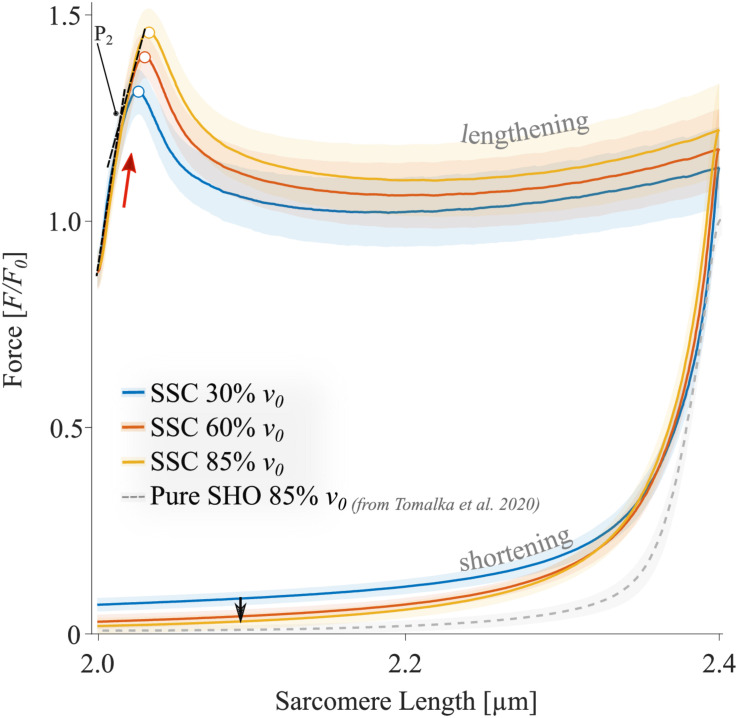
The mean ± standard deviation (SD) of force-length traces of SSC-contractions with varying velocities (control condition). Solid blue, red, and yellow lines indicate the means. The shaded regions around the solid lines indicate the corresponding SD during active SSCs (*n* = 14, raw data). Negative work was determined for a period of 1,190 ms (blue line), 650 ms (red line), and 450 ms (yellow line) during the lengthening phase from the onset of stretch until the end of stretch using numerical integration of force with respect to sarcomere length. Positive work was determined for equivalent periods during the shortening phase from the onset of release until the end of the release. The red arrow indicates the steep rise in force in the early phase of active muscle stretching during SSCs. The black arrow indicates the decrease in force with increasing shortening velocity (i.e., due to the *F-v*-r). The colored circles indicate the peak forces at the end of the stretch. The sarcomere length is recorded at maximal activation (*p*Ca 4.5) and is shown in μm. Additionally, pure shortening contractions of 2.4–2.0 μm with 85% *v*_0_ of rat soleus muscle fibers, derived from Tomalka et al. (2020), show forces that are obviously below the forces obtained during the shortening phase preceded by stretch (SSC).

The force-length traces of single muscle fibers are characterized by a steep rise in force in the early phase of active muscle stretching (red arrow in Figure 4), followed by a negative slope (up to about half the stretching time, Figure 4). Then the forces recovered relative to the initial drop in force to large parts toward the end of the stretch of the SSCs. The peak force during stretch increased with increasing stretch velocity (+10.7% ± 4.1%, *p* < 0.001, *d* = 2.49). Furthermore, maximum forces were reached at longer sarcomere lengths with increasing stretching velocity (+0.4% ± 0.2%, *p* < 0.001, *d* = 3.00; colored unfilled circles, Figure 4). During approximately 87% of the shortening phase, the highest forces were produced at the slowest shortening velocity (Figure 4, blue line).

### Blebbistatin Condition: Effects of XB-Inhibition on Work and Power as a Function of Velocity

**FIGURE 5:**
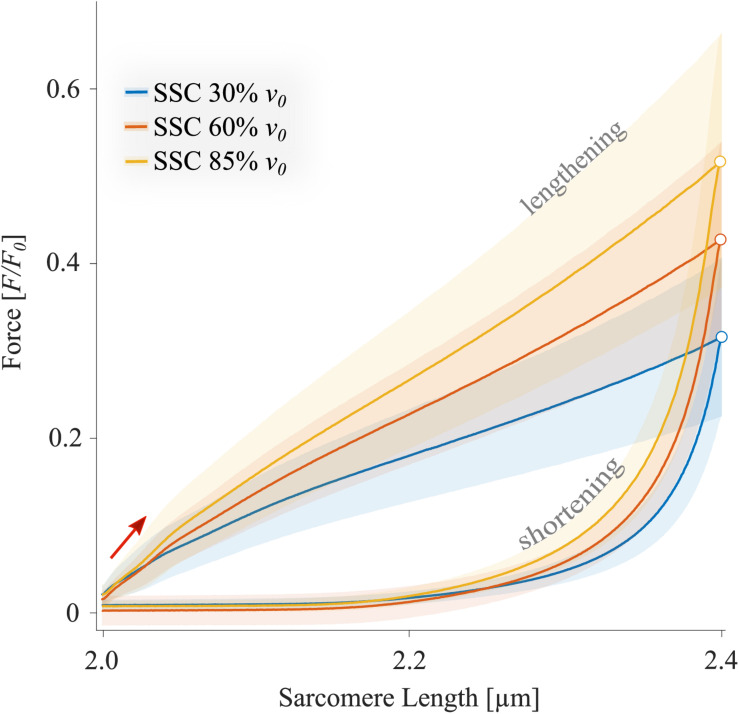
The mean ± standard deviation (SD) of force-length traces of SSC contractions with varying velocities (Blebbistatin condition). Blue, red, and yellow solid lines indicate the means. The shaded regions around the solid lines indicate the corresponding SD during active SSCs (*n* = 13, raw data). Negative work was determined for a period of 1,190 ms (blue line), 650 ms (red line), and 450 ms (yellow line) during the lengthening phase from the onset of stretch until the end of stretch using numerical integration of force with respect to sarcomere length. Positive work was determined for equivalent periods during the shortening phase from the onset of release until the end of the release. Peak forces at the end of the stretch are marked by colored circles in the presence of Blebbistatin.

The XB-inhibitor Blebbistatin decreased the maximum isometric forces to 0.02 *F*_0_ at 2.0 μm *L*_*S*_ (Figure 5). Accordingly, eccentric and concentric forces during the SSCs in the Blebbistatin condition were decreased (Figure 5) in comparison to the control condition (Figure 4). Maximum forces were reached at the end of the stretch and increased with increasing stretching velocity (colored circles, Figure 5).

**FIGURE 6:**
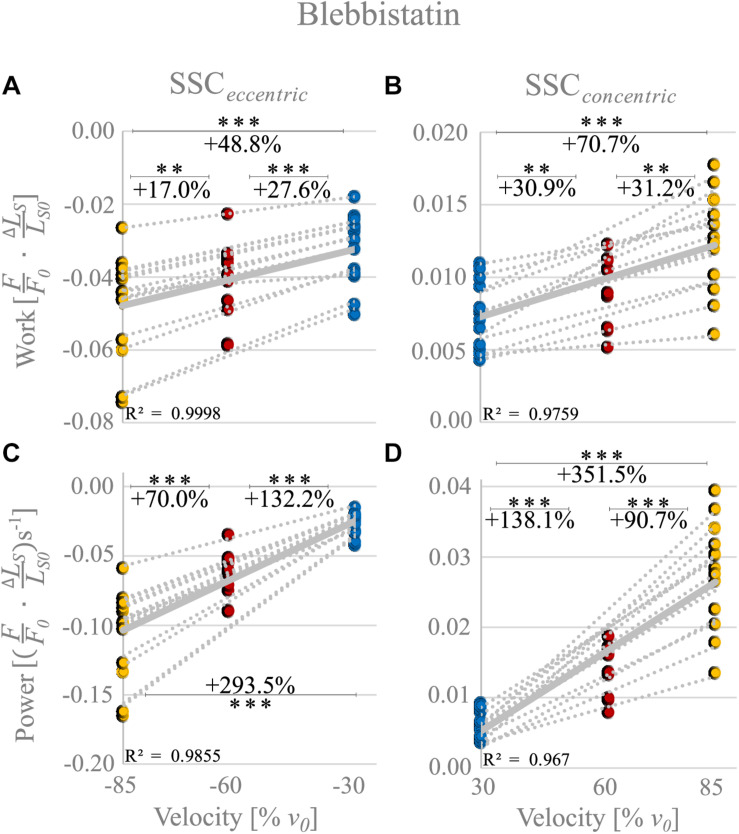
Blebbistatin condition. Influence of varying stretch-shortening velocities on work and power during the lengthening phase [SSC_*eccentric*_; negative work/power; **(A,C)**] and the shortening phase [SSC_*concentric*_; positive work/power; **(B,D)**] of SSCs, respectively. The gray dotted lines of the scatterplots shown in **(A–D)** indicate the individual paired data values, and solid gray lines indicate the mean values (*n* = 13 fibers from five rats). The blue circles indicate the SSC at (±)30% *v*_0_, the red circles at (±)60% *v*_0_, and the yellow circle at (±)85% *v*_0_. Statistical analyses are based on muscle fiber experiments in the presence of Blebbistatin. Work/power values are expressed in relative values $(\frac{F}{F_{0}}\cdot \frac{\Delta \,L_{S}}{L_{S\,0}}$) and $(\frac{F}{F_{0}}\cdot \frac{\Delta \,L_{S}}{L_{S\,0}}$)s^− 1^, respectively. Asterisks mark differences in work/power with varying velocities in the intergroup comparison. Significance levels are marked as follows: ^∗∗^*p* < 0.01 and ^∗∗∗^*p* < 0.001. *ns* means not significant. The Blebbistatin condition suggests the contribution of non-XB elements.

The negative work increased significantly with increasing velocities from slow to fast stretching velocities (+48.8% ± 12.4%, *p* < 0.001, *d* = 1.27, *R*^2^ = 0.99) during the stretching phase of the SSCs (cf. blue vs. yellow circles of Figure 6A and Table 2). Thus, negative work was significantly larger for fast compared with moderate velocities (+17.0% ± 7.61%, *p* < 0.01, *d* = 0.59; yellow vs. red circles of Figure 6A) and for moderate compared with slow stretching velocities (+27.6% ± 13.2%, *p* < 0.001, *d* = 0.83; red vs. blue circles of Figure 6A).

**TABLE 2:** Descriptive statistics and pairwise comparisons of work and power values obtained during the Blebbistatin experiments.

Blebbistatin								

				Pairwise comparisons work $[\frac{F}{F_{0}}\cdot$$\frac{\Delta \,L_{S}}{L_{\mathrm{S0}}}]$				*p*-values
	
Descriptive statistics				Mean differences	SD	95% Confidence interval of the difference		
		
Variable	Mean	SD				Lower	Upper	
W*_*ecc*_* 30% *v*_0_	−0.032	0.010	W*_*ecc*_* 30% *v_0_* −W*_*ecc*_* 60% *v*_0_	−0.008*	0.003	0.005	0.012	<0.001
W*_*ecc*_* 60% *v*_0_	−0.041	0.010	W*_*ecc*_* 60% *v_0_* −W*_*ecc*_* 85% *v*_0_	−0.007*	0.005	0.003	0.012	0.002
W*_*ecc*_* 85% *v*_0_	−0.048	0.014	W*_*ecc*_* 30% *v_0_* −W*_*ecc*_* 85% *v*_0_	−0.016*	0.006	0.010	0.021	<0.001
W*_*con*_* 30% *v*_0_	0.007	0.002	W*_*con*_* 30% *v_0_* −W*_*con*_* 60% *v*_0_	0.002*	0.001	−0.004	−0.001	0.004
W*_*con*_* 60% *v*_0_	0.010	0.002	W*_*con*_* 60% *v_0_* −W*_*con*_* 85% *v*_0_	0.003*	0.002	−0.005	−0.001	0.002
W*_*con*_* 85% *v*_0_	0.012	0.003	W*_*con*_* 30% *v_0_* −W*_*con*_* 85% *v*_0_	0.005*	0.002	−0.007	−0.003	<0.001

				**$\text{Power}[(\frac{F}{F_{0}}\cdot$$\frac{\Delta \,L_{S}}{L_{S\,0}})s^{1}]$**				

*P*_*ecc*_ 30% *v*_0_	−0.027	0.008	*P*_*ecc*_ 30% *v_0_* −*P*_*ecc*_ 60% *v*_0_	−0.035*	0.008	0.027	0.043	<0.001
*P*_*ecc*_ 60% *v*_0_	−0.062	0.016	*P*_*ecc*_ 60% *v_0_* −*P*_*ecc*_ 85% *v*_0_	−0.044*	0.017	0.028	0.061	<0.001
*P*_*ecc*_ 85% *v*_0_	−0.107	0.032	*P*_*ecc*_ 30% *v_0_* −*P*_*ecc*_ 85% *v*_0_	−0.079*	0.024	0.055	0.104	<0.001
*P*_*con*_ 30% *v*_0_	0.006	0.002	*P*_*con*_ 30% *v_0_* −*P*_*con*_ 60% *v*_0_	0.008*	0.002	−0.011	−0.006	<0.001
*P*_*con*_ 60% *v*_0_	0.015	0.004	*P*_*con*_ 60% *v_0_* −*P*_*con*_ 85% *v*_0_	0.013*	0.005	−0.018	−0.008	<0.001
*P*_*con*_ 85% *v*_0_	0.028	0.007	*P*_*con*_ 60% *v_0_* −*P*_*con*_ 85% *v*_0_	0.021*	0.006	−0.028	−0.015	<0.001

Work/power values ± SD are expressed in relative values $\left(\frac{F}{F_{0}}\cdot \frac{\Delta \,L_{S}}{L_{S\,0}}\right)$ and ($\frac{F}{F_{0}}\cdot \frac{\Delta \,L_{S}}{L_{S\,0}}$) s^–1^, respectively. *W*, mechanical work; *P*, power output; *ecc*, eccentric phase during active muscle stretch; *con*, concentric phase during active muscle shortening; %*v*_0_, percentage of maximum contraction velocity; *ns*, not significant. *The mean difference is significant at the 0.05 level.

For the shortening phase of the SSCs, the mechanical work increased significantly with increasing velocity (+70.7% ± 35.0%, *p* < 0.001, *d* = 1.74, *R*^2^ = 0.98, Figure 6B). Thus, positive work was significantly larger for moderate compared with slow velocities (+30.9%± 24.1%, *p* < 0.01, *d* = 0.89, Figure 6B) and for fast compared with moderate shortening velocities (+31.2% ± 18.4%, *p* < 0.01, *d* = 1.02). The overall negative work output during muscle stretch was about four times the amount of positive work during muscle shortening of SSCs.

The negative power output during muscle stretch of the SSCs increased significantly (+293.5% ± 32.7%, *p* < 0.001, *d* = 3.43, *R*^2^ = 0.99) with increasing stretching velocities (Figure 6C and Table 2). Negative power was significantly larger for fast compared with moderate stretching velocities (+70.0% ± 11.1%, *p* < 0.001, *d* = 1.78; Figure 6C) and for moderate compared with slow stretching velocities (+132.2% ± 24.0%, *p* < 0.001, *d* = 2.79; Figure 6C).

For the shortening phase of the SSCs, positive power output increased significantly (+351.5% ± 92.7%, *p* < 0001, *d* = 3.92, *R*^2^ = 0.97) with increasing shortening velocity. Power was significantly larger for moderate compared with slow shortening velocities (+138.1% ± 43.9%, *p* < 0.001, *d* = 2.89; Figure 6D) and for fast compared with moderate shortening velocities (+90.7% ± 26.7%, *p* < 0.001, *d* = 2.24; Figure 6D).

### Contributions of ‘Isolated XB’ Force to Work and Power as a Function of Velocity

**FIGURE 7:**
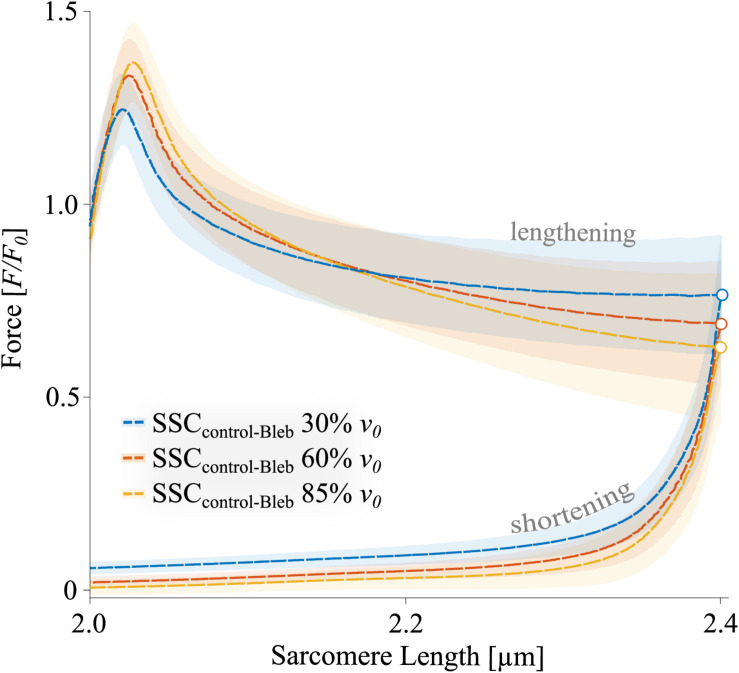
The mean ± standard deviation (SD) of force-length traces of ‘isolated XB’ forces in SSCs contractions with varying velocities. ‘Isolated XB’ forces were calculated by subtracting the inhibited forces of the Blebbistatin condition (Figure 5) from the total force responses during the SSCs of the control condition (Figure 4) (colored dashed lines, subtractions) (see section “Calculations of XB- and Non-XB Forces”). The shaded regions around the dashed lines indicate the corresponding SD during active SSCs. The colored circles indicate the peak forces at the end of the stretch.

To investigate the ‘isolated XB’ contributions to the total force response (Figure 7 and Table 3), Blebbistatin-suppressed forces (Figure 5) were subtracted from the total forces of control ramps (Figure 4) (see section “Calculations of XB- and Non-XB Forces”). Accordingly, differences in force, work, and power between control and Blebbistatin condition were referred to as ‘isolated XB’ forces, work, and power in the following.

**TABLE 3:** Descriptive statistics and pairwise comparisons of XB contributions to work and power.

Control minus Blebbistatin								

				Pairwise comparisons work $[\frac{F}{F_{0}}\cdot$$\frac{\Delta \,L_{S}}{L_{\mathrm{S0}}}]$				*p*-values
	
Descriptive statistics				Mean differences	SD	95% Confidence interval of the difference		
		
Variable	Mean	SD				Lower	Upper	
W*_*ecc*_* 30% *v*_0_	−0.163	0.015	W*_*ecc*_* 30% *v_0_* −W*_*ecc*_* 60% *v*_0_	0.000	0.006	−0.007	0.006	1.000
W*_*ecc*_* 60% *v*_0_	−0.163	0.014	W*_*ecc*_* 60% *v_0_* −W*_*ecc*_* 85% *v*_0_	0.001	0.005	−0.004	0.007	1.000
W*_*ecc*_* 85% *v*_0_	−0.164	0.017	W*_*ecc*_* 30% *v_0_* −W*_*ecc*_* 85% *v*_0_	0.001	0.006	−0.005	0.007	1.000
W*_*con*_* 30% *v*_0_	0.026	0.003	W*_*con*_* 30% *v_0_* −W*_*con*_* 60% *v*_0_	0.007*	0.002	0.005	0.009	<0.001
W*_*con*_* 60% *v*_0_	0.019	0.003	W*_*con*_* 60% *v_0_* −W*_*con*_* 85% *v*_0_	0.005*	0.002	0.003	0.006	<0.001
W*_*con*_* 85% *v*_0_	0.014	0.003	W*_*con*_* 30% *v_0_* −W*_*con*_* 85% *v*_0_	0.012*	0.002	0.010	0.014	<0.001

				**$\text{Power}[(\frac{F}{F_{0}}\cdot$$\frac{\Delta \,L_{S}}{L_{S\,0}})s^{1}]$**				

*P*_*ecc*_ 30% *v*_0_	−0.137	0.013	*P*_*ecc*_ 30% *v_0_* −*P*_*ecc*_ 60% *v*_0_	0.112*	0.011	0.100	0.123	<0.001
*P*_*ecc*_ 60% *v*_0_	−0.249	0.022	*P*_*ecc*_ 60% *v_0_* −*P*_*ecc*_ 85% *v*_0_	0.116*	0.017	0.099	0.133	<0.001
*P*_*ecc*_ 85% *v*_0_	−0.365	0.037	*P*_*ecc*_ 30% *v_0_* −*P*_*ecc*_ 85% *v*_0_	0.228*	0.025	0.202	0.254	<0.001
*P*_*con*_ 30% *v*_0_	0.022	0.003	*P*_*con*_ 30% *v_0_* −*P*_*con*_ 60% *v*_0_	−0.007*	0.002	−0.010	−0.005	<0.001
*P*_*con*_ 60% *v*_0_	0.029	0.004	*P*_*con*_ 60% *v_0_* −*P*_*con*_ 85% *v*_0_	−0.003	0.004	−0.006	0.001	0.611
*P*_*con*_ 85% *v*_0_	0.032	0.007	*P*_*con*_ 60% *v_0_* −*P*_*con*_ 85% *v*_0_	−0.010*	0.005	−0.015	−0.005	<0.001

XB contributions were calculated by subtraction of forces obtained during Blebbistatin condition from forces during control condition (section “Calculations of XB- and Non-XB Forces”). Work/power values ± SD are expressed in relative values $\left(\frac{F}{F_{0}}\cdot \frac{\Delta \,L_{S}}{L_{S\,0}}\right)$ and ($\frac{F}{F_{0}}\cdot \frac{\Delta \,L_{S}}{L_{S\,0}}$) s^–1^, respectively. *W*, mechanical work; *P*, power output; *ecc*, eccentric phase during active muscle stretch; *con*, concentric phase during active muscle shortening; %*v*_0_, percentage of maximum contraction velocity; *ns*, not significant. *The mean difference is significant at the 0.05 level.

**FIGURE 8:**
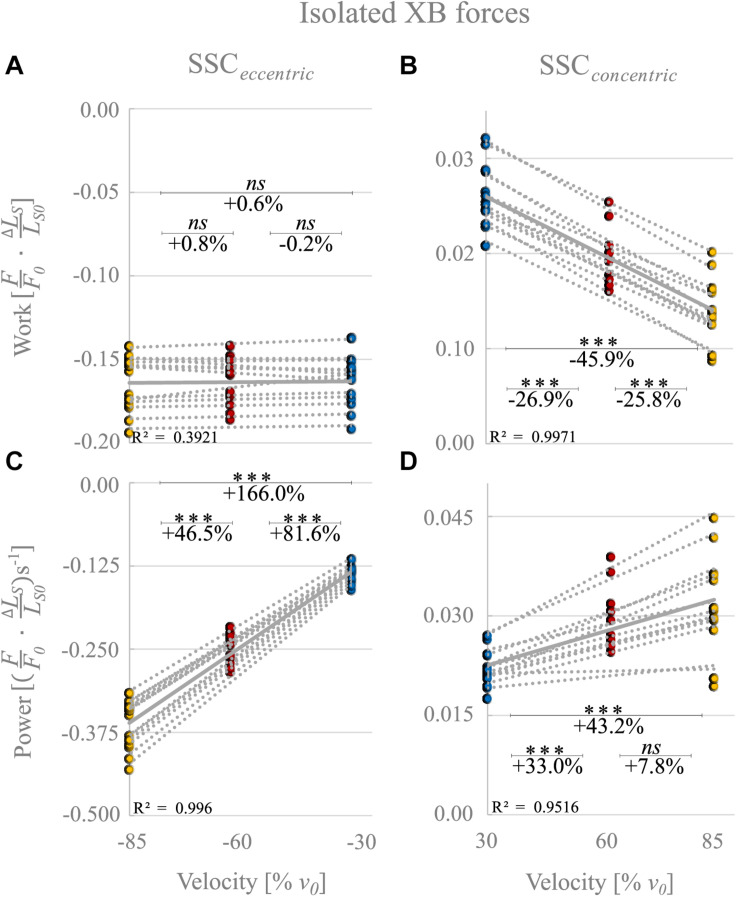
‘Isolated XB’ forces calculated by subtraction of forces obtained from control and Blebbistatin conditions. Influence of varying stretch-shortening velocities on work and power output during the lengthening phase **(A,C)** and the shortening phase **(B,D)** of SSCs, respectively. The gray dotted lines of the scatterplots shown in **(A–D)** indicate the individual paired data values, and gray solid lines indicate the mean values (*n* = 13 fibers from five rats). The blue circles indicate the SSC at 30% *v*_0_, the red circles at 60% *v*_0_, and the yellow circle at 85% *v*_0_. Statistical analyses are based on calculations of XB forces by subtraction of Blebbistatin-suppressed forces from control forces. Work/power values are expressed in relative values $(\frac{F}{F_{0}}\cdot \frac{\Delta \,L_{S}}{L_{S\,0}}$) and $(\frac{F}{F_{0}}\cdot \frac{\Delta \,L_{S}}{L_{S\,0}}$)s^− 1^, respectively. Asterisks mark differences in work/power with varying velocities in the intergroup comparison. Significance levels are marked as follows: ^∗∗∗^*p* < 0.001. *ns* means not significant.

‘Isolated XB’ forces reached at the end of the stretches decreased with increasing stretching velocity (colored circles, Figure 7). During the stretch phase of the SSCs (cf. blue vs. yellow circles of Figure 8A) negative work performed by ‘isolated XBs’ did not change with increasing velocities (+0.6% ± 3.9%, *p* = 1.00, *d* = 0.06, *R*^2^ = 0.39).

For the shortening phase of the SSCs, positive work of ‘isolated XBs’ decreased significantly with increasing velocity (−45.9% ± 7.6%, *p* < 0.001, *d* = 4.00, *R*^2^ = 0.99, Figure 8B). Thus, mechanical work of ‘isolated XBs’ was significantly smaller for moderate compared with slow velocities (−26.9% ± 5.0%, *p* < 0.01, *d* = 2.33, Figure 8B) and smaller for fast compared with moderate shortening velocities (−25.8% ± 9.6%, *p* < 0.01, *d* = 1.67).

The negative power output of ‘isolated XBs’ during muscle stretch of the SSCs increased significantly (+166.0% ± 10.4%, *p* < 0.001, *d* = 8.22, *R*^2^ = 0.99) with increasing stretching velocities (Figure 8C). Negative power of ‘isolated XBs’ was significantly larger for fast compared with moderate stretching velocities (+46.5% ± 4.6%, *p* < 0.001, *d* = 3.88; Figure 8C) and larger for moderate compared with slow stretching velocities (+81.6% ± 7.0%, *p* < 0.001, *d* = 6.20; Figure 8C).

For the shortening phase of the SSCs, the positive power output of ‘isolated XBs’ increased significantly (+43.2% ± 20.1%, *p* < 0001, *d* = 1.86, *R*^2^ = 0.95) with increasing shortening velocity. Power was significantly larger for moderate compared with slow shortening velocities (+33.0% ± 9.1%, *p* < 0.001, *d* = 1.98; Figure 8D). However, there was no change in positive power output of ‘isolated XBs’ for moderate compared with fast shortening velocities (+7.8% ± 13.9%, *p* = 0.611, *d* = 0.53; Figure 8D).

## Discussion

In contrast to our hypothesis, the power output during the shortening phase of the SSCs increased almost linearly (Figure 2, orange symbols) with increasing stretch-shortening velocity. This increase is contrary to the typical parabolic shape of the *P-v-*r for the same range of shortening velocities (Figure 2, orange solid line), which is based on the hyperbolic shape of the *F-v-*r (Hill, 1938). Accordingly, our main result does not comply with the cross-bridge theories of muscle contraction based on the interaction of the contractile proteins actin and myosin (Huxley, 1957). Based on the results from our experiments with the XB-inhibitor Blebbistatin, we suggest that the elastic protein titin plays a significant role in power output during SSCs. In the following, we discuss potential mechanisms that explain the increased power output during muscle shortening of SSCs.

### Control Condition: Influence of Muscle Fiber Kinetics on Mechanical Work and Power Output

#### Muscle Shortening

During the shortening phase of the SSCs, the positive work decreased (by 20%) with increasing velocities from 30 to 85% *v*_0_ (Figure 3B). The *F-v*-r can partly explain this decrease in work since forces decrease with increasing shortening velocity (Figure 2, blue line). Lower forces produced over the same shortening distance will result in decreasing work with increasing velocity. However, since power is work per unit of time, this ≈20% decrease in work is overcompensated by reducing the duration of the shortening phase (by 65%) from 30 to 85% *v*_0_. Thus, despite a decrease in work, the power output during the shortening phase significantly increased with increasing ramp velocities (Figure 3D).

#### Muscle Stretch

For all tested velocities in our study, fiber kinetics was characterized by a steep rise in force during the early phase of the stretch, immediately followed by a relatively compliant transient phase until the stretching has stopped. The initial linear phase (Figure 4, red arrow) is biphasic with a steep force slope followed by a more shallow slope (P_2_ transition; Figure 4). This observation is consistent with recent investigations of stretch-induced force responses (5% *L*_*S0*_ stretch amplitude) in intact and skinned muscle fibers (mammalian and amphibian) and over a wide range of velocities (Lombardi and Piazzesi, 1990; Burmeister Getz et al., 1998; Linari et al., 2003; Pinniger et al., 2006; Tomalka et al., 2020). Both force slopes mainly arise from XB characteristics and can be attributed to the extension of all attached myosin heads to actin (Pinniger et al., 2006). Remarkably, we observed a significant rightward shift of the initial force peak to longer muscle lengths with increasing velocity (Figure 4, unfilled colored circles), accompanied by an increased peak force (ranging from 1.3 to 1.45 *F*_0_). This rise in force after the P2 transition (≈1.5% *L*_*S0*_; Lombardi and Piazzesi, 1990) is attributed to the continuous stretch of non-XB elements (Edman and Tsuchiya, 1996; Pinniger et al., 2006; Roots et al., 2007). Thus, elastic energy stored in viscoelastic structures, such as titin, increases with increasing stretching velocity (Edman et al., 1978; Sugi and Tsuchiya, 1988; Pinniger et al., 2006).

Further, continuous muscle lengthening beyond P_2_, as investigated in this study, resulted in a negative force slope until the force recovers by the end of the stretching phase of SSCs. This characteristic transition phase has been referred to as muscle “give” (Flitney and Hirst, 1978). Muscle “give” is attributed to the detachment of myosin heads from thin filaments when the stretching velocity exceeds a particular threshold value that seems to be above 30% *v****_0_*** (Huxley, 1969; Sugi, 1972). However, the continuous rise in force after the previous “give” suggests the contribution of non-XB elements (Roots et al., 2007). This assumption is in line with previous work by Linari et al. (2003), who measured heat production and force of muscle fibers from frogs during ramp stretches. They suggested that XBs account for only ≈12% of the total energy storage during the active stretch. This amount includes the contribution of XB-elasticity (2.2% of total energy storage, Linari et al., 2000) and the redistribution of XB-states (≈9.8% of total energy storage, Linari et al., 2003), while XBs are pulled into states of higher energy during stretching.

Accordingly, more than 80% of energy storage cannot be explained by XB mechanisms, particularly since attached XBs detach quickly from actin filaments (Huxley and Simmons, 1971), and their stored elastic energy is lost (Bosco et al., 1982; Wilson et al., 1991). Consequently, non-XB structures, such as titin, may store and release most of the elastic energy during the SSCs’ eccentric and concentric phases, respectively, thereby increasing power output during the shortening phase (section “Blebbistatin Condition: Influence of Non-XB Structures on Mechanical Work and Power Output”).

### ‘Isolated XB’ Forces During the SSC

Based on the assumption that muscle force during a stretch is the sum of XB- and non-XB forces (Nocella et al., 2014; Tomalka et al., 2017, 2020), and that a high proportion of XB-based forces is switched off in the presence of Blebbistatin, subtraction of forces in the Blebbistatin condition from the forces in the control condition leads to XB-forces (Figure 7). Large parts of the stretch (≈2.1–2.4 μm *L*_*S*_) show XB forces clearly below 1.0 *F/F_0_*. This might be explained by muscle “give” since a fraction of XBs is torn off due to initial stretch. After the initial peak, the XB force continuously decreases until the end of the stretch for each stretch velocity (unfilled colored circles, Figure 7). Interestingly, in the second half of the stretch (2.2–2.4 μm *L*_*S*_), where an almost regular XB-cycling may be restored due to the constant stretching velocity, forces are lower (*p* < 0.001) for highest stretch velocity (85% *v*_0_, dashed yellow line, Figure 7) compared with the slowest stretch velocity (30% *v*_0_, blue dashed line, Figure 7). This contrasts with our typical understanding of the eccentric *F-v-*r, where forces increase with increasing negative velocities and plateau at a certain threshold (Joyce et al., 1969; Haeufle et al., 2014). One possible reason why this behavior is not yet mentioned in the literature might be that the decreasing XB contribution with increasing stretch velocity is overcompensated by an increasing non-XB contribution (Figure 5) to generate enhanced muscle fiber force as found in the control condition (Figure 4, second half of the stretch). However, this reasoning requires the basic assumption that Blebbistatin completely inhibits XB contributions to force production, which is further discussed below.

In the SSCs’ shortening phase, and in line with the *F-v-*r for shortening contractions (Hill, 1938), higher forces were produced at lower shortening velocity (30% *v*_0_, Figure 7, blue dashed line) compared with higher shortening velocity (85% *v*_0_, Figure 7, yellow dashed line). Consequently, the ‘isolated XB’-based work decreased with increasing shortening velocity (Figure 8B). However, the power produced by XBs increased with increasing shortening velocities (Figure 8D). This increase in power output can be explained by calculated XB forces (Figure 2, gray symbols) slightly above the *F-v-r* (Figure 2, blue line). Multiplication of these slightly higher forces with the respective shortening velocity results in the observed XB-based power output (*P* = *F* ×*v*) (Figure 8D). One possible explanation for the slightly enhanced XB forces (Figure 2, gray symbols) might be that too little non-XB forces were subtracted from control forces. Titin–actin interaction might be enabled (at least partially) by strong XB-binding (Leonard and Herzog, 2010; Powers et al., 2014; Tomalka et al., 2020). A reduced amount of strong XBs in the Blebbistatin condition might reduce non-XB-based forces and, thus, explains the slightly overestimated XB forces (Figure 2, gray symbols).

However, it cannot be taken for granted that Blebbistatin completely eliminates XB-based force production, since Blebbistatin [and similar drugs as butanedione monoxime (BDM) (Rassier and Herzog, 2004; Rassier, 2008) and benzyl-toluene sulfonamide (BTS) (Roots et al., 2007)] seems to affect the contractile apparatus in a complex manner (Minozzo and Rassier, 2010; Månsson et al., 2015). There are indications that Blebbistatin leads, among other things, to a considerable reduction of *v*_0_ under certain conditions (Stewart et al., 2009; Rahman et al., 2018). Furthermore, with Blebbistatin the relative force enhancement increases during the ramp stretch of 3–5% *L*_*S0*_ (Pinniger et al., 2006; Minozzo and Rassier, 2010). An effect that is explainable by the potential influence of an increased population of weakly bound XBs, which are suggested to contribute to an increase in stiffness and non-XB-based force while strained during muscle stretch (Pinniger et al., 2006; Rassier, 2008; Minozzo and Rassier, 2010; Rahman et al., 2018).

Regardless of the effect of Blebbistatin on the contractile apparatus, a contribution of weakly bound XBs to force during the stretch (Rassier, 2008; Minozzo and Rassier, 2010) seems to be likely only for small stretch amplitudes (≈1.5% *L*_*S0*_) (see section “Muscle Stretch”). For rather extensive ramp amplitudes of 17% *L*_*S0*_, as used in this study, weakly bound XBs rapidly detach (Schoenberg, 1985; Bagni et al., 2002). Thus, it seems unlikely that weakly bound XBs are primarily responsible for the observed significant peak forces at the end of the lengthening phase of the SSCs.

However, our approach does not clearly separate XB- and non-XB contributions. Accordingly, other inhibitors such as BTS or alternative approaches like the depletion of non-XB structures (e.g., selective digestion of titin by trypsin; Higuchi, 1992) should be considered for further studies attempting to separate XB- and non-XB contributions (Iwamoto, 2018; Ma et al., 2018).

### Blebbistatin Condition: Influence of Non-XB Structures on Mechanical Work and Power Output

Linari et al. (2003) observed an increase in energy storage with increasing stretch velocity in frog muscle fibers and suggested that non-XB structures may be responsible for this observation. Recently, there is increasing support that contributions of non-XB structures, such as titin, are (at least partially) responsible for the observed SSC-effect on the muscle fiber level (Fukutani et al., 2017b; Fukutani and Herzog, 2019, 2020b; Tomalka et al., 2020). It is essential to separate XB and non-XB contributions to total muscle force to approach the physiological mechanisms. Our Blebbistatin experiments, which suppress XB contributions, confirm these recent observations on the importance of non-XB structures for the stretch phase of SSCs and point to a velocity dependence of the SSC-effect in the control condition.

In the presence of Blebbistatin, when actin–myosin cycling is negligible, we found a quasi-linear force response during the SSCs’ stretch phase (Figure 5) with no muscle “give” upon active stretching (cf. section “Muscle Stretch”). Therefore, the increasing forces with increasing stretch velocities (Figure 5) point to higher loading of non-XB structures. This increased loading of titin or other non-XB structures with increasing stretch velocity contributes to increased energy storage during the stretch phase of SSCs, associated with amplified negative work and power (Linari et al., 2003; Pinniger et al., 2006; Tomalka et al., 2017).

Consequently, when a spring recoils, stored elastic energy is recovered in the shortening phase of SSCs and thus contributes to the observed performance amplification (Figures 3, 6), which was also suggested by Lindstedt (2016) and Hessel et al. (2017). However, significantly more work is done on the muscle upon active muscle stretching (negative work) than work generated by the muscle during the shortening phase of SSCs (positive work), typical for viscoelastic materials. Viscoelastic titin behavior during SSCs has been reported in previous studies (Bianco et al., 2007; Chung et al., 2011; Herzog et al., 2014) and may be attributed to titin’s mechano-structural properties. Thus, at low stretch velocities, temporary energy storage in viscoelastic elements leads to a significant reduction of muscle fiber’s negative power (Figures 3C, 6C), which agrees with Roberts and Azizi (2010).

Previous work showed that maximum eccentric forces in the XB-inhibited conditions are enhanced by 11-fold than passive experiments (without Blebbistatin and calcium) at 85% *v*_0_ (Tomalka et al., 2020). This enhancement is partly attributed to calcium-induced stiffening of titin (by 20%) during activation (Labeit et al., 2003). However, more important seems to be the property of skeletal muscle titin to significantly reduce its persistence length upon activation, potentially by titin–actin interactions (Herzog, 2014; Nishikawa, 2016). As a beneficial consequence, the reduced titin length allows for increased force production during subsequent stretching (Kellermayer and Granzier, 1996; Labeit et al., 2003; Rode et al., 2009; Dutta et al., 2018; Tahir et al., 2020). These mechanisms might increase non-XB forces (presumably titin-based) during the stretch and enable the muscle to raise the total energy storage during active muscle stretching. Although many previous studies have investigated titin–actin interactions in the muscle (Kellermayer and Granzier, 1996; Astier et al., 1998; Nagy, 2004; Bianco et al., 2007; DuVall et al., 2017; Dutta et al., 2018; Li et al., 2020; Powers et al., 2020), naming of a target site for such interactions would be speculative at this time. For further information on the description of physiological mechanisms for the increase of titin stiffness in the active muscle, we refer to recent reviews (Linke, 2017; Herzog, 2018; Nishikawa, 2020).

### Energy Recovery

The energy recovery (W*_*con*_*/W*_*ecc*_*; calculated by dividing the work output obtained during the shortening phase of the SSC by the work done during the lengthening phase) significantly decreases in the control condition with increasing velocity (from 17.4% at 30% *v*_0_ to 12.8% at 85% *v*_0_). This decrease is partly due to the XBs’ dissipative properties, as dissipation increases with velocity. Thus, inhibiting XB-cycling by Blebbistatin leads to an increase in energy recovery. The ratio (W*_*con*_*/W*_*ecc*_*) is higher in the Blebbistatin condition compared with the control experiments. Interestingly, in the Blebbistatin condition, the energy recovery (W*_*con*_*/W*_*ecc*_*) tends to increase slightly with increasing velocity (from 23.9% at 30% *v*_0_ to 26.4% at 85% *v*_0_, *ns*). This increase suggests that titin can be mechanically understood as a spring in series with a damper (Millard et al., 2019; Brunello and Fusi, 2020; Powers et al., 2020). For such a spring-damper system, at a higher velocity, due to the serial damper, the spring is stretched with higher force (and can store more energy in the spring element) and thus release more energy when it is shortened. Although the force at the end of the stretch in the Blebbistatin condition is only about a third of the force in the control experiments (cf. Figure 4 vs. Figure 5), the work during the XB-inhibited shortening phase of SSCs (at 85% *v*_0_) reached nearly 50% of the work reported in the control condition. This finding implies a comparatively high energy recovery by titin.

### Implications for *in vivo* Muscle Action

Despite clear evidence of the SSC-effect across all structural muscle levels (for a recent review, see Groeber et al., 2019), the contraction modalities (such as, e.g., the stretch-shortening amplitude and contraction velocity) might have important implications on experimental findings of comparable studies in the literature. In general, there are conflicting results regarding the occurrence of active SSCs in the muscle fascicles themselves during *in vivo* human movements. Depending on movement tasks studied, SSCs without (Cronin and Finni, 2013; Lai et al., 2015; Aeles and Vanwanseele, 2019) and with fascicle stretch (Ishikawa et al., 2005; Rubenson et al., 2012; Nikolaidou et al., 2017) have been reported. To date, no mechanism exclusively explains the SSC-effect and there likely is an interaction of mechanisms at different structural levels, with a dominance, e.g., depending on involved muscle (group) or movement dynamics. The transfer of experimental *in vitro* findings on *in vivo* SSC-effects should be considered with caution; however, the existence of an SSC-effect on the fiber level cannot be neglected. Since many (cyclical) everyday movements occur at submaximal muscle activation levels (Groeber et al., 2019), future studies should be done in skinned muscle fibers at different calcium concentrations to better understand the meaning of the SSC-effect in *in vivo* situations.

## Conclusion

In the present study, we found work and power amplification in the shortening phase of SSCs in control- and Blebbistatin conditions. Interestingly, (i) this SSC-effect is velocity-dependent since the power output increases with increasing velocity. (ii) The energy recovery (ratio of elastic energy storage and release in the SSC) is higher in the Blebbistatin condition compared with the control condition. This amplified energy recovery in the Blebbistatin condition can be explained by the viscoelastic properties of the non-XB structure titin.

This SSC-effect study promotes a basic understanding of human locomotion since SSCs are part of the most basic, everyday-type of muscle contraction. The separation of XB- and non-XB structures is of primary importance to give a more detailed understanding of the potential involvement of viscoelastic elements, such as titin, working as an energy-storing spring during lengthening contractions and SSCs. This information is required for the improvement of muscle models (Heidlauf et al., 2016; Tahir et al., 2018; Seydewitz et al., 2019) as well as for improved predictions by multi-body models (Röhrle et al., 2017; Haeufle et al., 2020) concerning, e.g., movement control and efficiency of locomotion.

## Data Availability Statement

The original contributions presented in the study are included in the article. Further inquiries can be directed to the corresponding author/s.

## Ethics Statement

The skeletal muscle fibers from rats used for this study have been provided by another animal study that was approved according to the regulations of the German Animal Protection Law (Tierschutzgesetz, §4 (3); Permit Number: 35-9185.81/0491) by the Regierungspräsidium Stuttgart, Department of Landwirtschaft, Ländlicher Raum, Veterinär- und Lebensmittelwesen.

## Author Contributions

AT, TS, DH, and WS contributed to the conceptualization of the study and edited and revised the manuscript. SW and AT performed the experiments. AT analyzed the data, prepared the figures, and drafted the first version of the manuscript. AT and TS analyzed and discussed the results. All authors contributed to the article and approved the submitted version.

## Conflict of Interest

The authors declare that the research was conducted in the absence of any commercial or financial relationships that could be construed as a potential conflict of interest.

## Abbreviations

ATP: adenosine 5 ′ triphosphate disodium salt hydrate
CK: creatine phosphokinase
Ca^2+^: calcium
CP: creatine phosphate
E-64: *trans*-epoxysuccinyl-L-leucylamido(4-guanidino)butane
EGTA: ethylene glycol-bis(2-aminoethylether)-N,N,N′,N′-tetraacetic acid
*F/F_0_*: maximum isometric muscle force
*F*-*l*-r: force-length-relation
*F*-*v*-r: force-velocity-relation
rFD: residual force depression
rFE: residual force enhancement
GLH: glutathione
HDTA: 1,6-diaminohexane-N,N,N′,N′-tetraacetic acid
*h*: height
IMID: imidazole
KOH: potassium hydroxide
KP: potassium propionate
*L/L_0_*: optimum muscle fiber length associated with *F/F_0_*
*L/L_*S0*_*: optimum sarcomere length associated with *F/F_0_*
*L*_*S*_: individual sarcomere length
non-XB: non-cross-bridge
*P*_0_: maximum power output
PMSF: phenylmethanesulfonyl fluoride
*P*-*v*-r: power-velocity-relation
SSC: stretch-shortening cycle
TES: *N*-[Tris(hydroxymethyl)methyl]-2-aminoethanesulfonic acid
v/v: volume/volume
*v*_0_: maximum shortening velocity
w/v: weight/volume
*w*: width
XB: cross-bridge.
